# Supplementary material for: Planar Junctionless Field-Effect Transistor for Detecting Biomolecular Interactions
Source: Sensors (Basel). 2022 Aug 2;22(15):5783. doi: 10.3390/s22155783 (PMC9371156; doi:10.3390/s22155783)
Supplement: Supplementary file 1 [file sensors-22-05783-s001.zip › sensors-1835958-supplementary.pdf]

Planar junctionless field-effect transistor for detecting biomolecular interactions

Rajendra P. Shukla [a*], Johan Bomer [a], Daniel Wijnperle [a], Naveen Kumar [b], Vihar P. Georgiev [b], Aruna Chandra Singh [d], Sivashankar Krishnamoorthy [d] and César Pascual García [c], Sergii Pud [a*], and Wouter Olthuis [a*]

[a] BIOS Lab-on-a-Chip Group, MESA+ Institute for Nanotechnology, University of Twente & Max Planck Centre for Complex Fluid Dynamics, Enschede, 7500 AE, the Netherlands
[b] Device Modelling Group, School of Engineering, University of Glasgow, Glasgow G12 8LT, UK
[c] Nano-Enabled Medicine and Cosmetics Group, Materials Research and Technology Department, Luxembourg Institute of Science and Technology (LIST), Belvaux, L-4362, Luxembourg
[d] Nanoscale Engineering for Devices & Bio-Interfaces, Nanotechnology Unit of the Materials Research and Technology Department, Luxembourg Institute of Science and Technology (LIST), Belvaux L-4422, Luxembourg
e-mail: r.p.shukla@utwente.nl, s.pud@utwente.nl

1. Electrical characterization and Annealing temperature Optimization:

Electrical characterization of the device was done using a Semiconductor parameter analyzer (Keithley, 4200A system) and a probe station attached to it. After annealing, the I-V (two terminal current-voltage) characteristics of the devices were measured to test the ohmic behavior. Electrical characterization of the first batch of the fabricated FETs devices was done after annealing at 450 ^{0}C for 30 minutes (following ref. [1]) and it was found that the devices show nonlinear I-V characteristics measured between source and drain contacts of the device (Figure S1A). It was found that the annealing temperature plays a big role in electrical contact behavior in these devices due to the formation of different silicide compounds at different annealing temperature and time [2–4]. To find out the optimized annealing temperature, metal patterns were evaporated on silicon wafer with similar resistivity and orientation as for SOI wafers. The Ta/Pt patterned wafer was diced in to several chips for annealing temperature optimization. The samples were annealed at 200, 250, 300, 350, 400, 450, 500 ^{0}C for 15 minutes and then I-V characteristics were recorded for all the samples. Figure S1B shows the I-V characteristics of all the samples annealed at different temperatures which shows a linear I-V characteristics with different slopes related to different electrical resistance values. Figure S1C and S1D shows the extracted resistance values from these I-V characteristics and plotted as a function of annealing temperature. It is evident from these figures that the resistance value is found to be lowest for 300 and 350 ^{0}C annealing temperatures. For lower and higher annealing temperatures than these the resistance value was increased which is due to the formation of different compounds of silicide adding extra layer in between Pt and Si at the interface that increases the resistance of the contacts. To further explore this phenomenon of silicide formation at different temperatures, the annealed samples were etched in aqua regia solution. Aqua regia etches only platinum not silicide (silicide are found in different forms; e.g. Pt_3Si, Pt_2Si, PtSi, $PtSi_2$, $PtSi_3$).

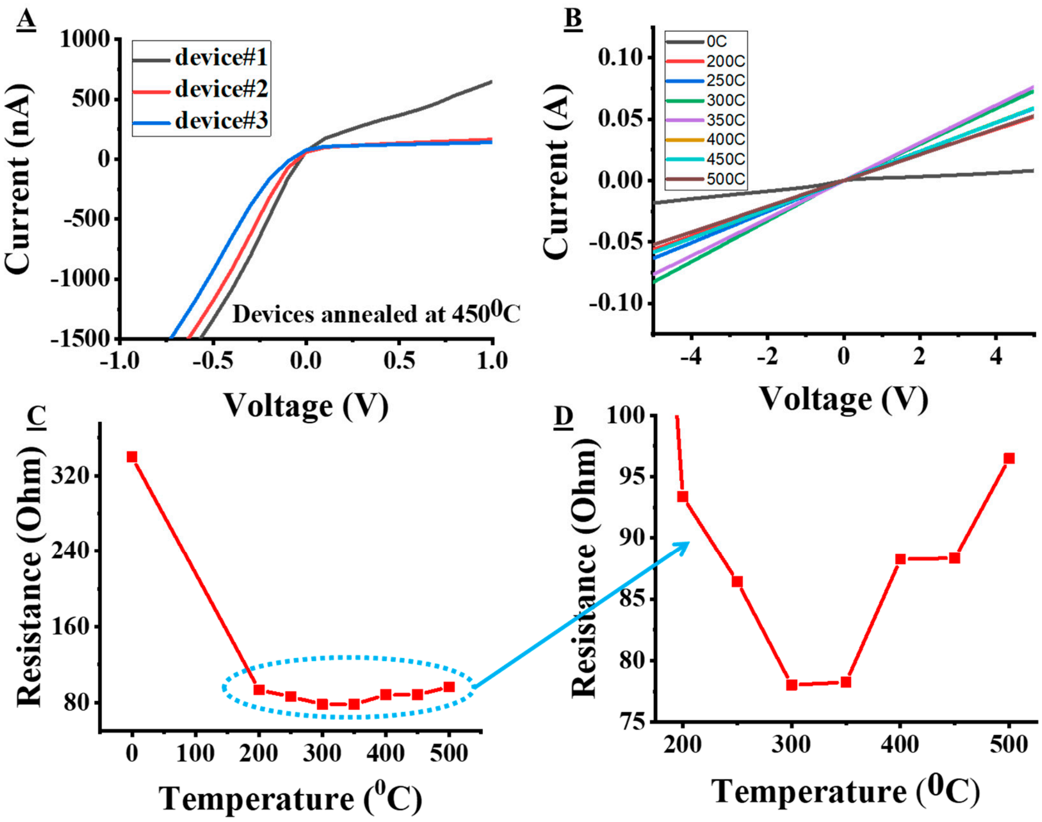

Figure S1. Annealing optimization of contacts. (A) Electrical characterization of the devices on SOI wafer annealed at 450^{0}C, (B) Electrical characterization of the metal patterns on Si wafers at different annealing temperatures, (C) and (D) resistance vs. annealing temperature.

When the sample was etched and the profile was measured with dektak profiler, a height profile was observed due to silicide formation in platinum (Figure S2). The silicide formation happens in both ways as silicon and platinum both are consumed in their respective proportions depending on the annealing temperature. The physics of different compounds of silicide formation is rather a complex phenomenon which is not the scope of this manuscript and reported elsewhere [3–5]. It is found in literature that the silicide formation of the Pt-Si interface starts at around ~200 ^{0}C(it is debatable as different literature reports different values of temperature for silicide formation, some reporting the start at 100 ^{0}C, others at 300 ^{0}C) [5]. The height profile of these samples indicates that the surfaces with lower annealing temperatures which is the starting of the silicide formation, the roughness is higher (can be seen from error bars of the current values in S2). At 350 ^{0}C the roughness is lowest and for other temperatures the rate of silicide formation increases and that is why we have higher thickness of the silicidized interface. For an ohmic contact, the silicon should be in direct contact with the high work function of the Pt. Thin layer of silicide is formed when annealing is done and is playing positive role in ohmic contact behavior due to intermediate work function of silicide (with respect to Pt and Si), However, presence of thick silicide in between Pt and Si degrades the ohmic behavior. The higher thickness of silicide adds more resistance to the contacts.

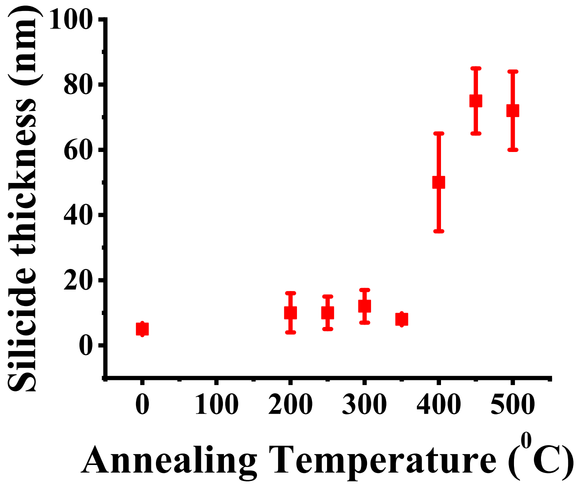

Figure S2. Height profile measurement of the Pt-etched samples annealed at different temperatures showing the height profile of the silicide formations.

1.1. Surface characterization:

To check the surface of the silicide, SEM images were taken. Figure S3 shows the SEM images of the etched metal patterns on the silicon wafer annealed at different temperatures 200, 350 and 500^{0}C. Figure S3A,B shows the low and high resolution SEM images of the etched metal patterns after annealing. This shows that at lower temperature where the formation of the silicide starts, the surface is rough and the silicide formation is non-uniform. Figure S3C shows the SEM image of the interface of the Pt and Si which also clearly indicate the presence of silicide at lower temperatures. For annealing temperature of 350 ^{0}C, the surface is less rough as compared to low temperature annealing and it looks more uniform (Figs. S3D, S3E, and S3F). At 500 ^{0}C, the pores on the surface can be seen that shows the higher thickness and presence of more silicide on the silicon surface (Figs. S3G, S3H, and S3I). These pores are adding more roughness to the interface. At higher annealing temperatures the pores of the silicide can be seen whereas at moderate temperatures the surface uniformity of silicide formation can be seen. The better surface coverage of the silicide formation at 350 ^{0}C is the reason for lower surface roughness as seen in height profile measurement. From the above optimization it is found that the annealing temperature of 350 ^{0}C has shown low roughness and a uniform interface as compared to other annealing temperatures. Therefore, it was decided to proceed with 350 ^{0}C as annealing temperature for SOI wafers. It was also observed that the presence of oxygen is one of the most important factors in the silicide formation. If there is oxygen, the electrical resistance was found to be higher due to the diffusion of oxygen through platinum forming another oxide compound at Pt-Si interface.

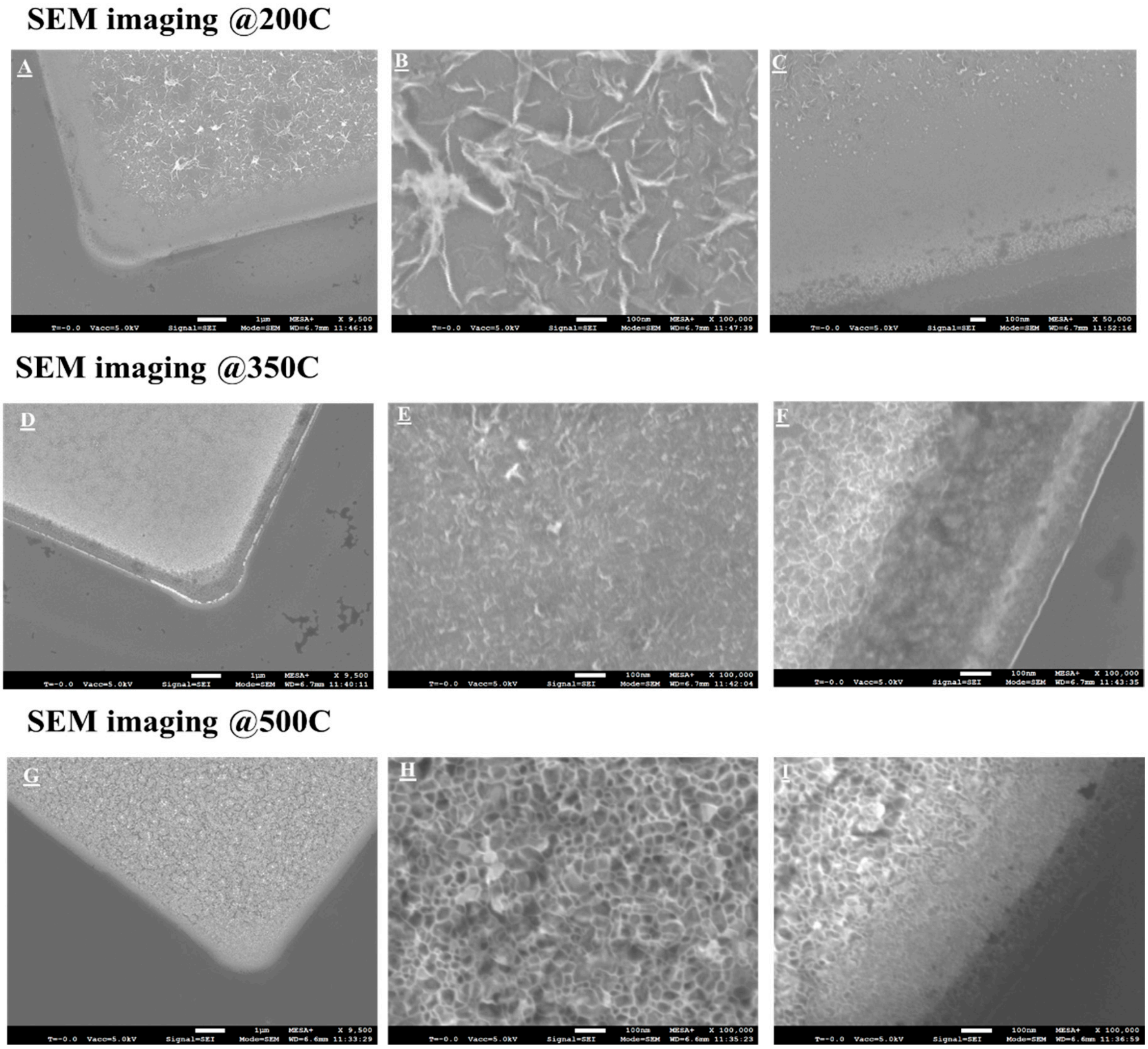

Figure S3. Scanning electron microscopy of the silicide surface annealed at 200^0C (A) low and (B) high resolution image, and (C) interface of Pt-Si. For annealing temperature of 350^0C, (D) low and (E) high resolution image and (F) interface of Pt-Si. For annealing temperature of 500^0C, (G) low and (H) high resolution image and (I) interface of Pt-Si.

Following the results of the annealing optimization, new batch of planar junctionless FETs devices were fabricated on SOI wafer. The two-terminal (measured between source and drain) electrical characterization of these devices again shows a non-linear I-V characteristics. Figure S4A shows the non-linear I-V characteristics of the FETs devices on SOI wafer annealed at 200 and 350 ^{0}C. By further exploring the physics of the devices and processes involved for the fabrication, it is found that there is another phenomenon taking place and that is the out diffusion of the Boron dopant atoms during the long hours of oxidation to thin down the device layer [6]. This increases the overall resistivity of the devices.

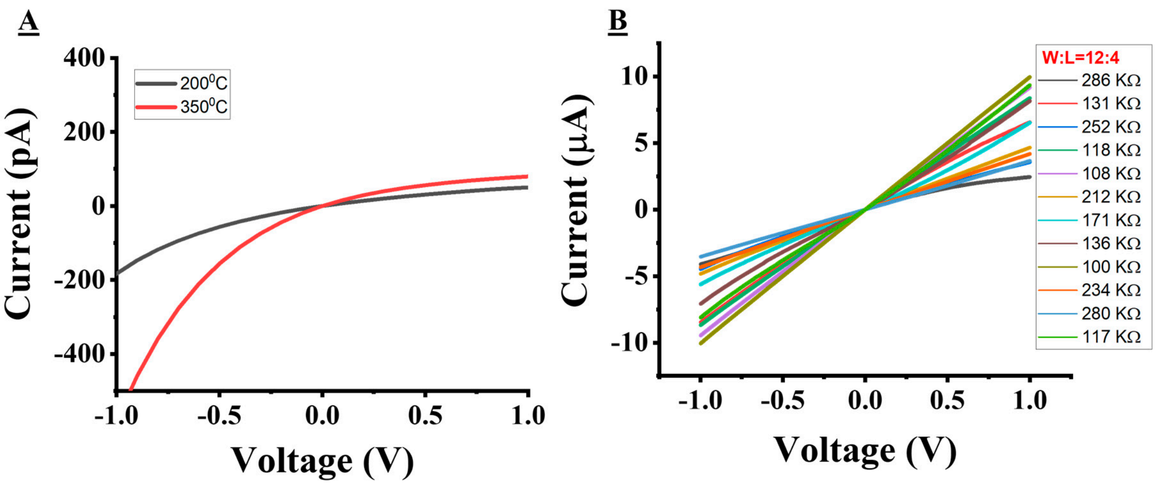

Figure S4. (A) Electrical I-V characteristics of the FETs device, and (B) Electrical I-V characteristics of the FET device with source and drain doped with boron impurity using PECVD process followed by drive in.

To address this challenge the source and drain regions were doped with Boron after thinning down the device layer. The process flow was revised and devices were fabricated on SOI wafers by doping the source and drain regions. The electrical characterization of these devices was done after annealing at 350^{0}C for 15 minutes and it shows a linear I-V characteristics (Figure S4B). However, it has shown larger variability in I-V characteristics among the devices. The reason behind this was further investigated and it was found that the variability in I-V behavior is coming from the variability in device layer thickness which is 0.5 µm and our working device layer thickness lies in the variance range of the overall device layer thickness (250-300 nm). Sometimes, it is wise to check the I-V characteristics of the device while using a liquid gate with defined potential as the open gate area may introduce some unmeasurable potential shift to the gate voltage due to charging and discharging.

1.2. I-V characterization planar junctionless FETs device with Optimized process protocol:

The devices with doping source and drain regions were characterized after annealing temperature optimization. The process flow was revised and devices were fabricated on SOI wafers by doping the source and drain regions and using nitride as a mask layer to avoid unnecessary etching of BOX layer which is adding problem in metal surface coverage at the contacts. The electrical characterization of these devices was done after annealing at 350^{0}C for 15 minutes and it shows linear I-V characteristics. Figure S5 shows the I-V characteristics of the junctionless FETs devices which shows a linear response.

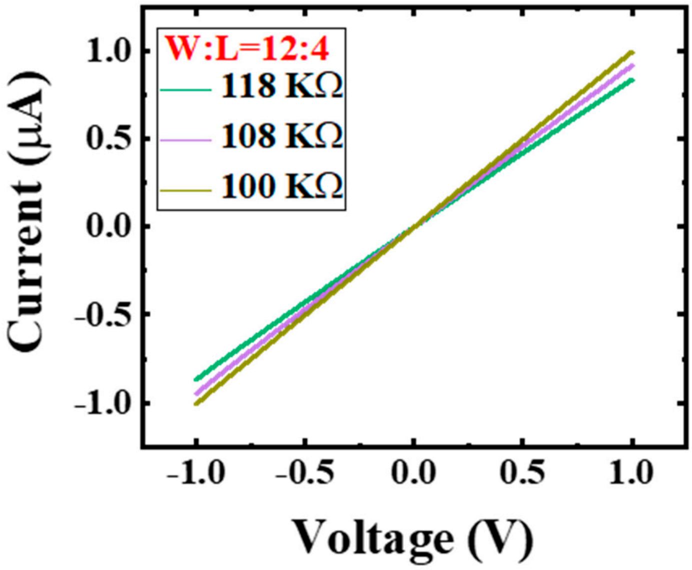

Figure S5. I-V characteristics of the FETs devices with doping source and drain regions.

1.3. Calculating surface charge density:

The current vs. charge density curve is extracted from the Ref. gate bias variation with respect to pH value as shown in Figure 6 in the manuscript. As ref. gate bias follows the surface potential for a constant current, the surface potential vs. pH characteristics was extracted by solving the Gouy-Chapman-Stern and Site-Binding model simultaneously while assuming the same isoelectric point (pH_{iso}=2) for different current values. Due to a constant value of stern capacitance (0.8 F/m^2), potential at the shear plane (zeta potential) was calculated. Thereafter, the surface charge density was calculated using the zeta potential and pH as the variable parameters for the Gouy-Chapman-Stern model which results in the same charge density calculated from the site-binding model with surface potential and pH as the variable parameters. This approach confirms and justifies the same charge density (opposite sign) of the electrical double layer while acting as a correction check of the proposed model. The different slope of obtained Ref. gate bias vs pH curves results in similar behavior for the obtained surface charge density values(Figure S6) [5].The obtained current vs charge density sensitivity can help in correlating the charge variation on FET surface due to protein-peptide interactions.

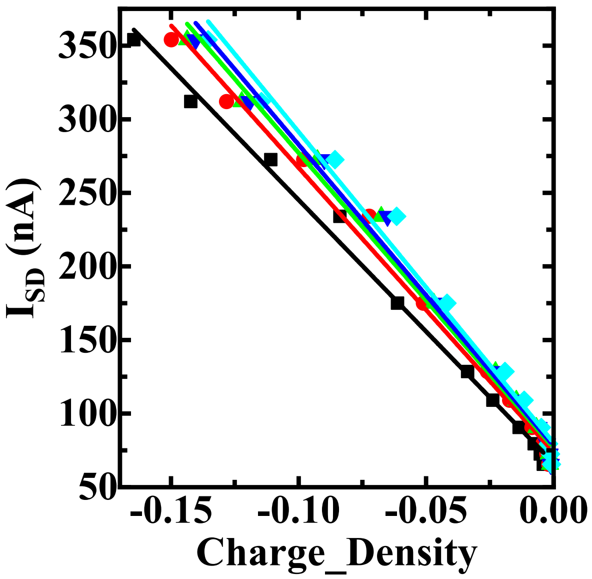

Figure S6. Drain current vs. surface charge density curve of the experimental data and linear fit extracted from the ref. Gate bias vs. pH graph with different current values (Figure 4C in Manuscript).

2. Surface functionalization of SiO_2 with APTES, Peptide and proteins:

We have functionalized the SiO_2 with different layers that will be combined with the FETs SiO_2 surface. In this manuscript, we are showing it to compare the surface groups present in FETs due to SiO_2 are comparable with the APTES functionalized SiO_2 surface. These two things will later be combined which is not the scope of this manuscript.

2.1. Surface preparation and functionalization for Surface plasmon resonance (SPR) investigation:

The Au/SiO_2 surface was grafted with a peptide, and used to study its binding with specific protein of interest. Figure S7 illustrates the different steps to functionalize the Au/SiO_2 surface with the peptide and its interaction with an antibody. As a first step, the Au/SiO_2 SPR sensor is functionalized with aminopropyl trimethoxy silane (APTES) in vapor phase to form a self-assembled monolayer (SAM) on the surface. The APTES functionalized SPR sensor was then exposed to a hetero bifunctional crosslinker 3-succinimidyl-3-maleimidopropionate (SMP) to introduce maleimide groups. The maleimide functionalized SPR sensor was subsequently used to graft peptides (A peptide with 16 amino acid sequence, with a cysteine at its C-terminal). The choice of cysteine enables -SH groups that can be used to crosslink the peptides with maleimide functionalized sensor surface. Any unreacted maleimide groups are blocked by exposure to m-PEG-thiols in excess.

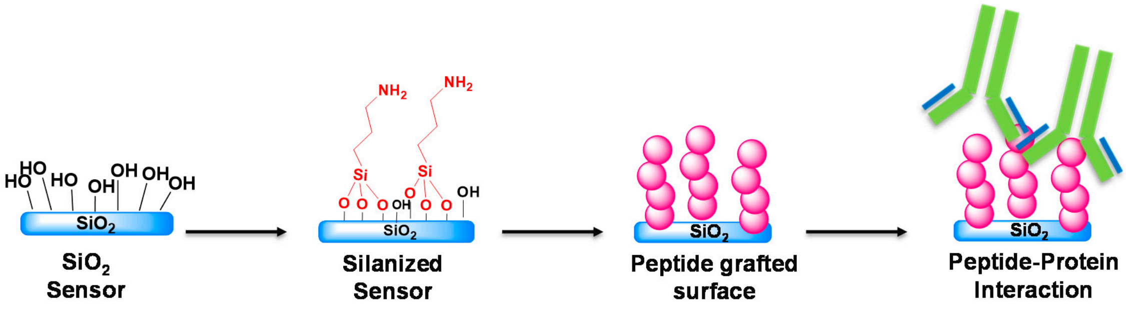

Figure S7. Schematic representation of the different steps of the Au/SiO_2 surface of the SPR sensor to study peptide-protein interactions.

2.2. Surface characterization using XPS:

The XPS spectra were recorded with Kratos Axis Ultra DLD instrument using a monochromatic aluminum source (Al Kα 1486.6 eV) at an operating voltage of 150 W. Three different spots for analysis were chosen with an elliptical area of 700 µm x 300 µm. All elemental spectra were acquired at pass energy = 160 eV and 20 eV to get comprehensive information on oxidation states and chemical structure. Table S1 shows the elemental and chemical composition of the analyzed sensor chip before and after APTES functionalization. The elemental analysis confirms the adsorption of APTES on surface by the presence of primary amine of APTES and additional peaks (Showed in Figure S8 and S9).

Table S1. Elemental and chemical composition recorded with XPS on bare SiO_2, and APTES grafted SiO_2 sensor.

Elemental composition					
**Sample**	**C%**	**N%**	**O%**	**Si 2p%**	
Bare SiO_2	6.9	0.0	63.3	26.3	
SiO_2+APTES	34.2	7.1	38.1	11.1	
Chemical composition					
**Sample**	**SiO_2**	**O=C**	**SiOC**	**Si4+**	**Si3+**
Bare SiO_2	83	0	0	100	0
SiO_2+APTES	11	21	34	19	81

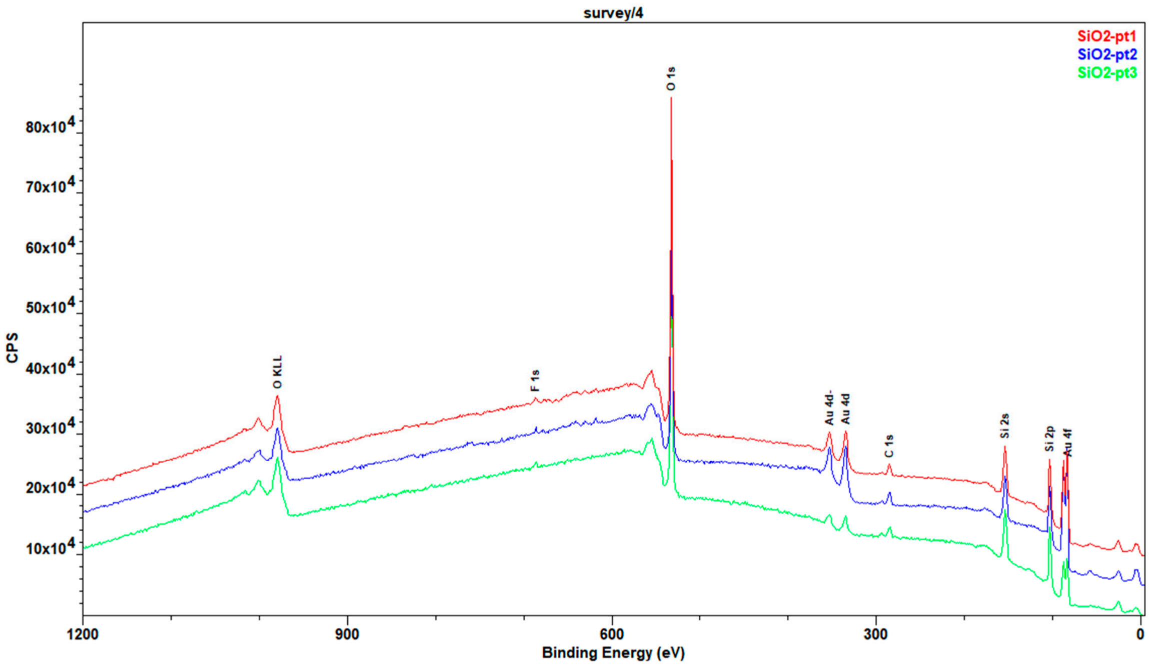

Figure S8. XPS spectra showing the presence of elements on bare SiO_2 sensor

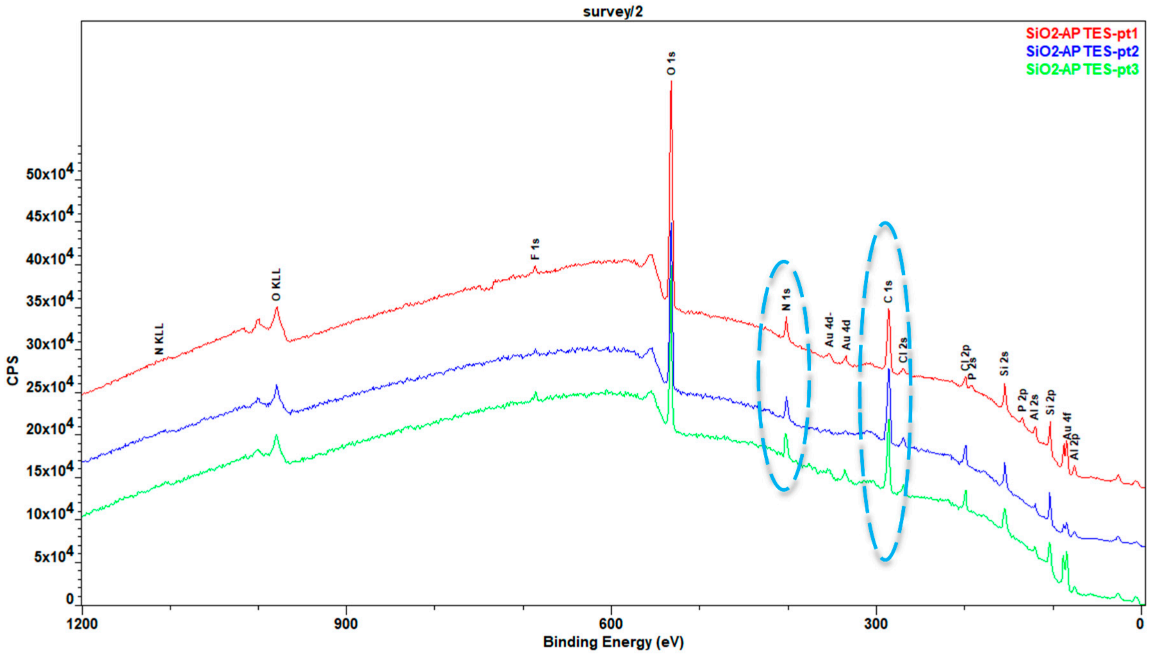

Figure S9. XPS spectra showing the presence of elements on APTES grafted SiO_2 sensor

The shape of the O 1s peak (Figure S10 (a)) is changing according to the distribution and bonding on the surface between 531 eV and 534 eV (SiOC peak at 532.65 eV±1.5 eV attributed to APTES). The peak at 103.4 eV is denoting Si 2p (Figure S10 (b) which was assigned to Si atom bounded to oxygen and carbon in the APTES molecule. The nitrogen N 1s core level (Figure S10 (c)) exhibited two peaks, one located at 400.4 eV which is related to amine group

bounded to carbon C-NH_2. The second peak located at 402.3 eV could be attributing to C-NH_3+.

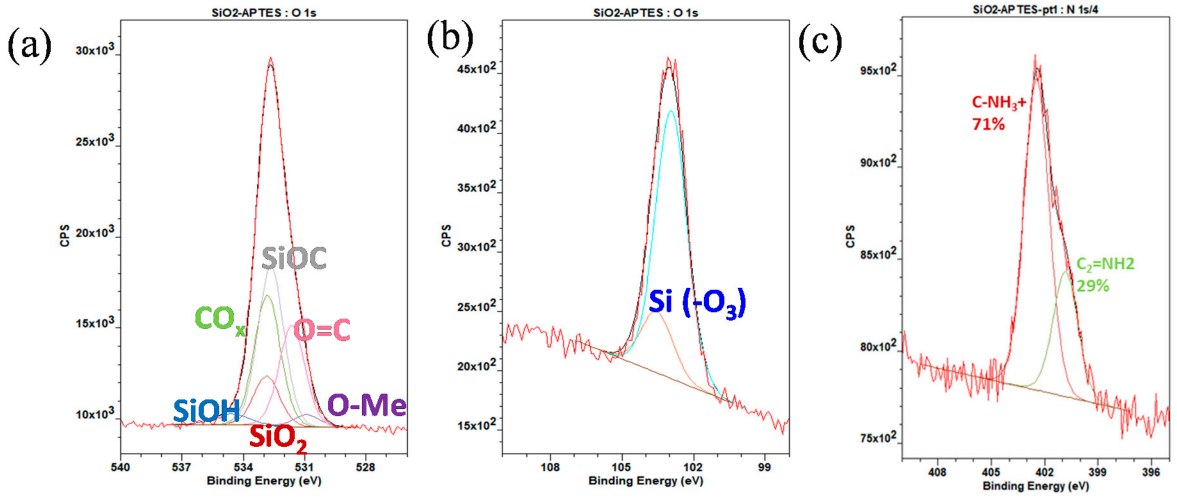

Figure S10. XPS spectra for Silicon and primary amine presence arrangement after APTES grafting on bare SiO_2 sensor.

2.3. Measuring APTES functionalization using SPR:

Functionalization of the Au/SiO2 SPR chips with APTES was performed ex-situ. The reflectance change due to APTES functionalization was measured in air, by taking the difference in the angle of the reflectance dip, before and after the APTES immobilization. This process is efficient to have APTES density of $1.3x10^{15}$ molecules on the sensor surface left which corresponds to the presence of monolayer of APTES.

2.4. Monitoring peptide-protein interactions on SPR:

SPR was used to monitor in-situ immobilization of the peptide onto the Au/SiO2 surface, as well as the interaction of peptide with a specific polyclonal antibody (Figure S11). The peptide solution (1000 ng/mL or 560 nM) was flowed onto SMP functionalized Au/SiO2 SPR surface at a flow rate of 10 µL/min, for 20 min. The peptide was injected twice to get a molecular density of $2.8x10^{13}$ molecules/cm^2. The unreacted sites of SMP were blocked using m-PEG-thiol to avoid non-specific binding. The peptide grafted sensor surface was exposed to different concentrations of specific primary antibody (pAb) in the concentration range of 0.1-50 µg/mL, which resulted in binding densities of 7 - 994 ng/cm^2 (Table 1 in the main manuscript).

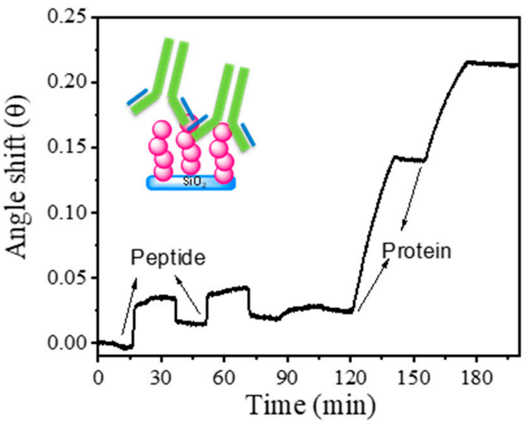

Figure S11. Representative SPR response showing real time binding events of peptide and Ab (1μg/mL).

3. Drift analysis in Phosphate Buffer solution:

As a control experiment, we have carried out the experiment for drift analysis in phosphate buffer solution (PBS, pH 7.74). The calculated drift was found to be -6 nA/hour which shows lower drift as compared to the buffer solution used in the paper as per Figure 6B ((% ΔI_{ds}/hour = 6% for TBACl and 0.2% for phosphate buffer solution)). This signifies the buffer solution dependence of drift in junctionless FETs.

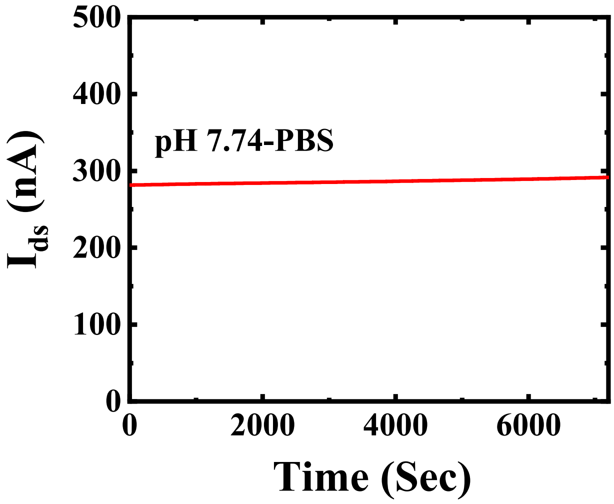

Figure S12. I_{ds} vs. time for a pH of 7.74 PBS buffer

1. Gueye, R., et al., *Fabrication and formation of Ta/Pt-Si ohmic contacts applied to high-temperature Through Silicon Vias (TSVs).* Sensors and Actuators a-Physical, 2013. **191**: p. 45-50.
2. Poate, J.M. and T.C. Tisone, *Kinetics and Mechanism of Platinum Silicide Formation on Silicon.* Applied Physics Letters, 1974. **24**(8): p. 391-393.
3. Cohen, S.S., et al., *Platinum Silicide Ohmic Contacts to Shallow Junctions in Silicon.* Journal of Applied Physics, 1982. **53**(12): p. 8856-8862.

4. Naem, A.A., *Platinum Silicide Formation Using Rapid Thermal-Processing.* Journal of Applied Physics, 1988. **64**(8): p. 4161-4167.
5. Faber, E.J., R.A.M. Wolters, and J. Schmitz, *On the kinetics of platinum silicide formation.* Applied Physics Letters, 2011. **98**(8).
6. Grove, A.S., *Physics and technology of semiconductor devices*. 1967, New York,: Wiley. xix, 366 p.
